# Evolving trend change during the COVID-19 pandemic

**DOI:** 10.3389/fpubh.2022.957265

**Published:** 2022-09-20

**Authors:** Liping Gao, Canjun Zheng, Qi Shi, Kang Xiao, Lili Wang, Zhiguo Liu, Zhenjun Li, Xiaoping Dong

**Affiliations:** ^1^State Key Laboratory of Infectious Disease Prevention and Control, National Institute for viral Disease Control and Prevention, Chinese Center for Disease Control and Prevention, Beijing, China; ^2^Chinese Center for Disease Control and Prevention, Beijing, China; ^3^State Key Laboratory of Infectious Disease Prevention and Control, National Institute for Communicable Disease Control and Prevention, Chinese Center for Disease Control and Prevention, Beijing, China

**Keywords:** COVID-19, epidemics evolve, geographic distribution, variants, vaccine

## Abstract

Coronavirus disease (COVID-19) has caused unimaginable damage to public health and socio-economic structures worldwide; thus, an epidemiological depiction of the global evolving trends of this disease is necessary. As of March 31, 2022, the number of cases increased gradually over the four waves of the COVID-19 pandemic, indicating the need for continuous countermeasures. The highest total cases per million and total deaths per million were observed in Europe (240,656.542) and South America (2,912.229), despite these developed countries having higher vaccination rates than other continents, such as Africa. In contrast, the lowest of the above two indices were found in undeveloped African countries, which had the lowest number of vaccinations. These data indicate that the COVID-19 pandemic is positively related to the socio-economic development level; meanwhile, the data suggest that the vaccine currently used in these continents cannot completely prevent the spread of COVID-19. Thus, rethinking the feasibility of a single vaccine to control the disease is needed. Although the number of cases in the fourth wave increased exponentially compared to those of the first wave, ~43.1% of deaths were observed during the first wave. This was not only closely linked to multiple factors, including the inadequate preparation for the initial response to the COVID-19 pandemic, the gradual reduction in the severity of additional variants, and the protection conferred by prior infection and/or vaccination, but this also indicated the change in the main driving dynamic in the fourth wave. Moreover, at least 12 variants were observed globally, showing a clear spatiotemporal profile, which provides the best explanation for the presence of the four waves of the pandemic. Furthermore, there was a clear shift in the trend from multiple variants driving the spread of disease in the early stage of the pandemic to a single Omicron lineage predominating in the fourth wave. These data suggest that the Omicron variant has an advantage in transmissibility over other contemporary co-circulating variants, demonstrating that monitoring new variants is key to reducing further spread. We recommend that public health measures, along with vaccination and testing, are continually implemented to stop the COVID-19 pandemic.

## Introduction

Coronavirus disease (COVID-19), which is caused by infection with severe acute respiratory syndrome coronavirus (SARS-CoV-2), has had a devastating impact on global health, security, and economies, including forcing people to find alternative ways of working, traveling, and communicating (1). COVID-19 was first reported in East Asia, and then rapidly spread to Europe, North America, South America, Africa, and Oceania, causing several waves of pandemics in different regions (2, 3). Despite no strict definition for a pandemic wave, a pandemic wave is considered to consist of an increasing number of sick individuals, a clearly defined peak in this number, and finally a decline (4, 5). Moreover, a previous predictive model for COVID-19 explored the behavior of a pandemic wave, which can provide vital clues for policymakers to design tailored action plans (6).

When the World Health Organization (WHO) first declared the COVID-19 pandemic a Public Health Emergency of International Concern on January 1, 2020, all nations worldwide began to take action (7). Despite the adoption of similar containment measures, the number of confirmed cases and mortality rates largely differed among countries (8). As of March 31, 2022, COVID-19 has affected 225 countries and territories, and 490,071,097 cases of COVID-19 have been recorded, with 6,158,664 deaths. Viruses mutate or change their genetic material following replication, which serves to create variants (9). In the case of SARS-CoV-2, the same mutation has emerged independently in different countries, indicating its potential benefit for viral fitness (10, 11). The variants of SARS-CoV-2 are categorized as variants of interest (VOI) or as variants of concern (VOC) by the WHO Virus Evolution Working Group. VOCs have increased transmissibility compared to that of the original virus and have the potential to increase disease severity (12). SARS-CoV-2 infections remain a leading cause of morbidity and mortality and have triggered an unprecedented number of global health researchers and scientists to work to develop safe and effective vaccines to reduce the spread and severity of infection (13). Today, multiple highly effective vaccines have been developed and are being administered in countries worldwide, providing several clinically evaluated and approved therapeutic options (14). As of March 31, 2022, 64.6% of the global population has received at least one dose of the COVID-19 vaccine; 11.33 billion doses have been administered globally, and 15.55 million are now administered daily. However, only 14.7% of people in low-income countries have received at least one vaccine dose (https://ourworldindata.org/covid-vaccinations). In this study, we aimed to use the open and freely available data related to COVID-19 published online from around the world to explore changes in pandemic trends across six continents, and to provide valuable insight to better understand the epidemiological evolution of COVID-19. This will help decision-makers implement tailored strategies to contain further spread of the disease.

## Methods

### Ethics statement

This study was supported by the China–Sierra Leone Biosafety Laboratory Technical Cooperation Project (III Phase) and was approved by the Commission of Ethics and Science Censor of the Sierra Leone Ministry of Health and Sanitation. Our survey adhered to the medical ethics of domestic laws and regulations.

### Data source, process, and definition of a wave

In our study, data relating to epidemiological indexes (e.g., cases and deaths) and related social factors, as well as SARS-CoV-2 variants from each country worldwide, were extracted from global public COVID-19 surveillance websites, including OurWorldInData.org (https://ourworldindata.org/), and Gisaid.org (https://www.gisaid.org/). Subsequently, all of the epidemic indices and available items were extracted and processed by month, including the number of cases, number of deaths, total cases per million, total deaths per million, and diversity profile of SARS-CoV-2 globally and across six continents; these items had been originally processed by day in the abovementioned databases. All of the data were cross-checked by two trained qualified health workers. The acquired data were then cleaned and analyzed using Microsoft Excel (Microsoft Office 2016, Microsoft Corporation, Redmond, WA, US). Furthermore, the death rate was calculated as follows: death rate = deaths /confirmed cases × 100%. In this study, we used the study variable “pandemic wave” (hereafter referred to as wave), which was defined as the time from the start of a peak (first month with increasing numbers of cases) to the end of a peak (month with a nadir of cases before the next rise). The waves were classified as follows: the first wave (wave 1), July 2020 to February 2021, and Pre-wave 1, January to June 2020; the second wave (wave 2), March 2021 to June 2021; the third wave (wave 3), July 2021 to October 2021; and the fourth wave (wave 4), November 2021 to March 2022.

## Results

### Epidemiology profile of COVID-19 worldwide

**Figure 1:**
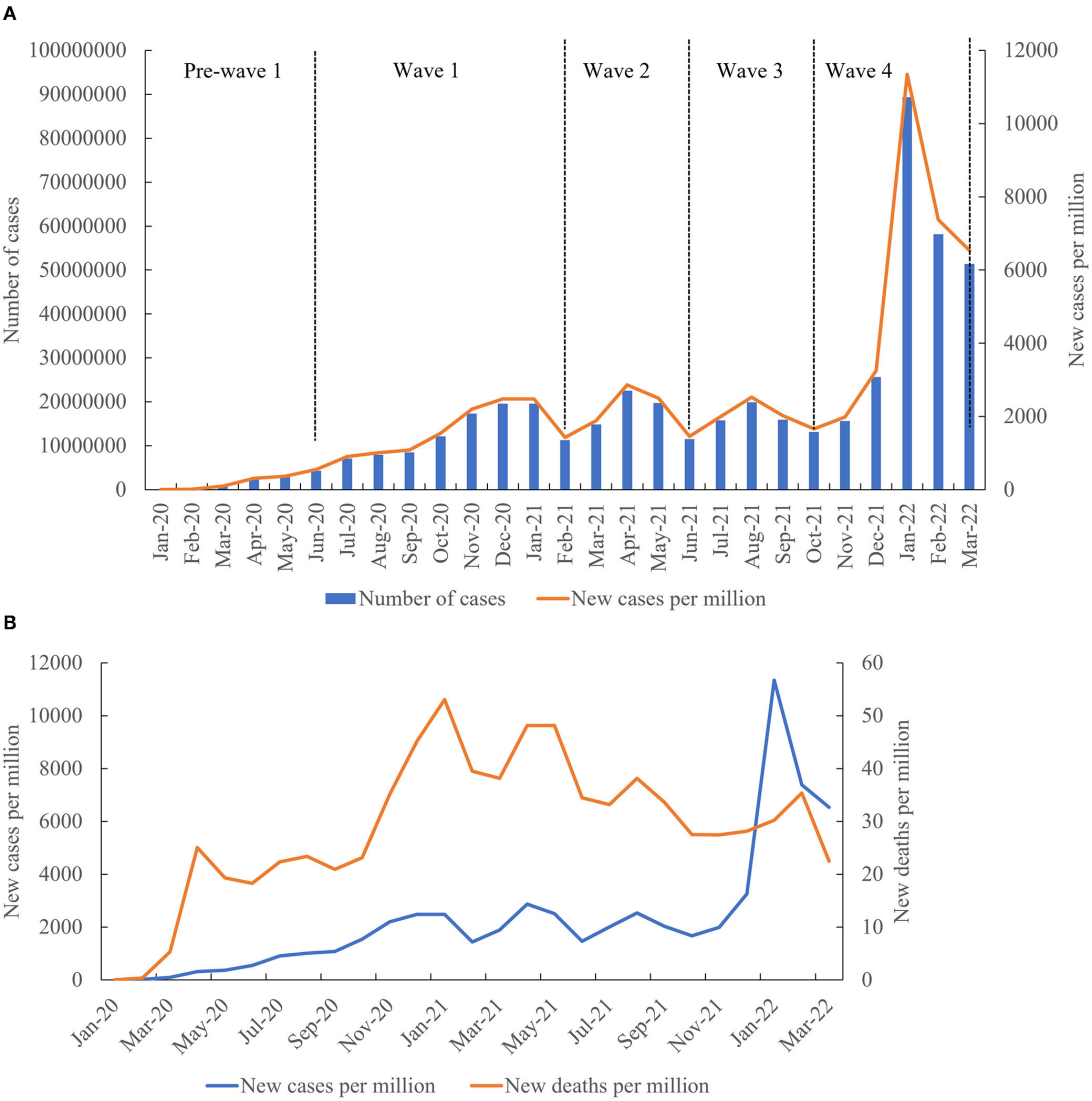
Epidemiology profile of COVID-19 in the six continents. **(A)** Epidemiological trends in the six continents. **(B)** Evolution of cases and deaths per million in the six continents over time.

**Figure 2:**
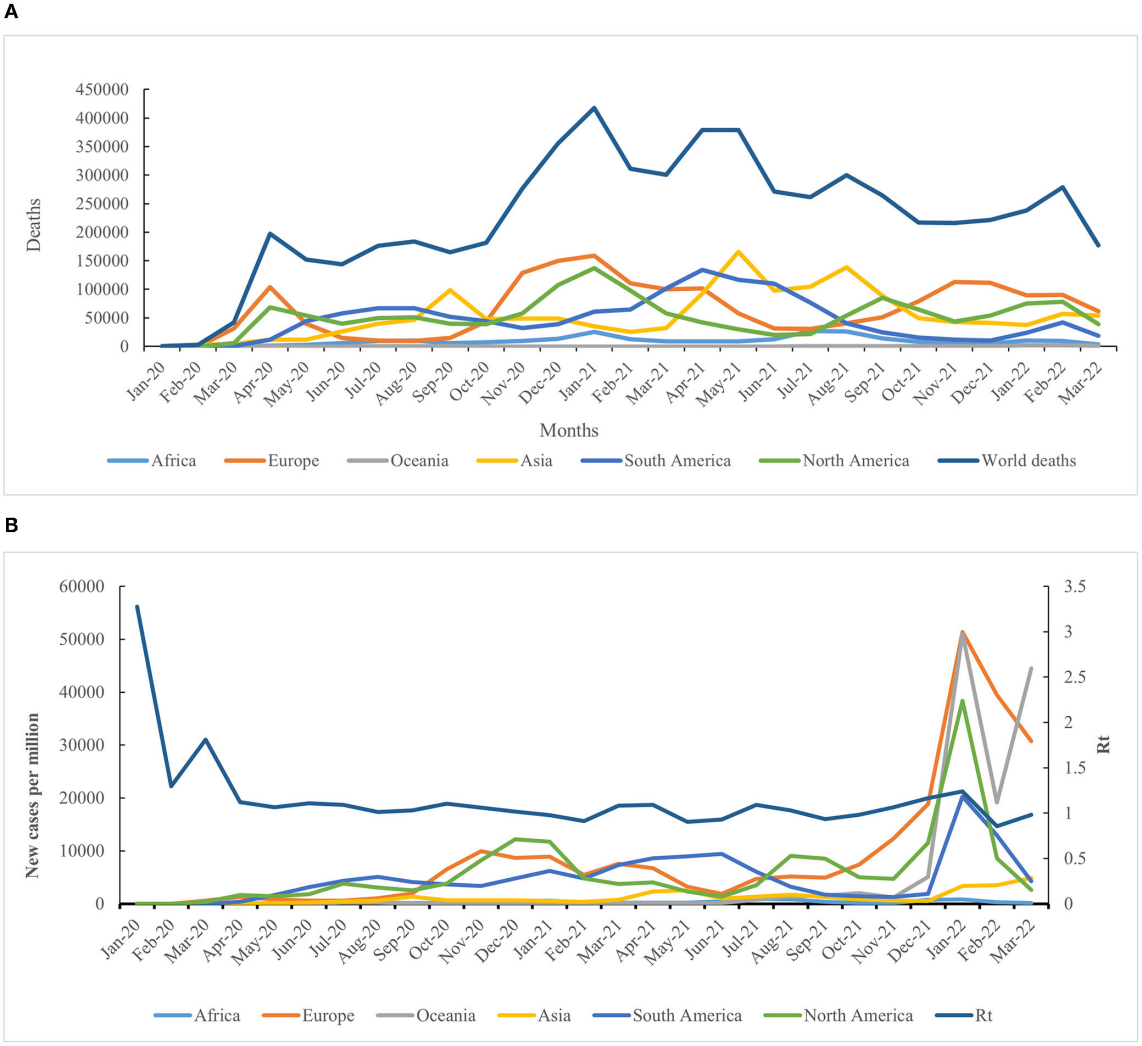
Geographic distribution features of deaths and Rt value. **(A)** Evolution of COVID-19-related deaths in the six continents over time. **(B)** Dynamic fluctuation of the Rt value in the six continents among the four waves of the COVID-19 pandemic.

By March 31, 2022, a total of 490,071,097 cases of COVID-19 had been recorded, with 6,158,664 deaths globally (Figures 1A and 2A) (WHO). The total cases per million and total deaths per million were 61,876.916 and 775.723, respectively (Figure 1B). The COVID-19 pandemic comprises four notable global waves during this period: the first, from January 2020 to February 2021; the second, from March 2021 to June 2021; the third, from July 2021 to October 2021; and the fourth, from November 2021 to March 2022 (Figure 1A). The number of confirmed cases was 116,657,428 in the first wave, 68,577,450 in the second wave, 64,667,001 in the third wave, and 240,169,218 in the fourth wave. The fourth wave was the highest, accounting for 49.01% (240,169, 218/490, 071,097), which was approximately four-fold greater than that of the lowest wave (the third wave). Based on the month, the highest case numbers (*n* = 89,374,078) were reported in January 2022, while the lowest was recorded in January 2020 (*n* = 9,370), with approximately 18,150,781 cases per month on average (Figure 1A). The confirmed cases in Europe and North America were much higher in the first wave; those in Asia were much higher in the second and third waves; those in Europe and Asia were much higher in the fourth wave. Although the number of cases in the fourth wave increased exponentially compared to the first three waves, the number of deaths experienced an increasing trend in the first wave and a declining trend in the fourth wave (Figure 1B). The number of deaths in waves 1–4 was 2,655,110, 1,330,041, 1,042,864, and 1,130,649, respectively, with the highest number of deaths observed in the first wave and the lowest in the third wave. Additionally, the highest death number (*n* = 417,837) was noted in January 2021, while the lowest was observed in January 2020 (*n* = 196) (Figure 2A).

### Geographic distribution features of cases and deaths in six continents

Both confirmed and fatal cases were reported in six continents, all of which experienced a four-wave COVID-19 pandemic. The numbers of confirmed COVID-19 cases from low to high were 5,547,963 in Oceania, 11,541,119 in Africa, 55,959,246 in South America, 94,616,144 in North America, 142,163,779 in Asia, and 180,242,846 in Europe. Accordingly, the number of deaths from low to high was 8,925 in Oceania, 251,740 in Africa, 1,264,662 in South America, 1,410,089 in North America, 1,450,615 in Asia, and 1,772,633 in Europe (Figure 2A).

The highest number of positive and fatal cases were recorded in Europe, whereas the lowest incidence was reported in Oceania (Figure 2A). As of March 31, 2022, the crude fatal rate (CRF) worldwide was 1.25%, with a rate of 2.18% in Africa, 1.00% in Asia, 0.98% in Europe, 0.16% in Oceania, 1.50% in North America, and 2.30% in South America. As time progressed, the CRF was reduced, except for in South America, where it increased by 11% (Supplementary Table S1).

The highest incidence rate based on the cases per million was observed in Europe (*n* = 240,656.542), and the highest death rate based on the deaths per million was observed in South America (*n* = 2,912.229), while the lowest incidence and death rates were reported in Africa (8,402.799 and 183.269). The incidence rate in other regions, from high to low, was 158,597.247 in North America, 128,861.1 in South America, 128,365.784 in Oceania, and 30,386.972 in Asia. Furthermore, the total deaths per million in other regions, from high to low, was 2,366.794 in Europe, 2,363.603 in North America, 310.065 in Asia, and 206.492 in Oceania (Figure 2B). The reproduction rate (Rt) of COVID-19 reduced sharply in April 2020, then tended to be stable, and finally showed a fluctuating decrease during the fourth wave (Supplementary Figure S1).

### Diversity and distribution profile of SARS-CoV-2 variants

**Figure 3:**
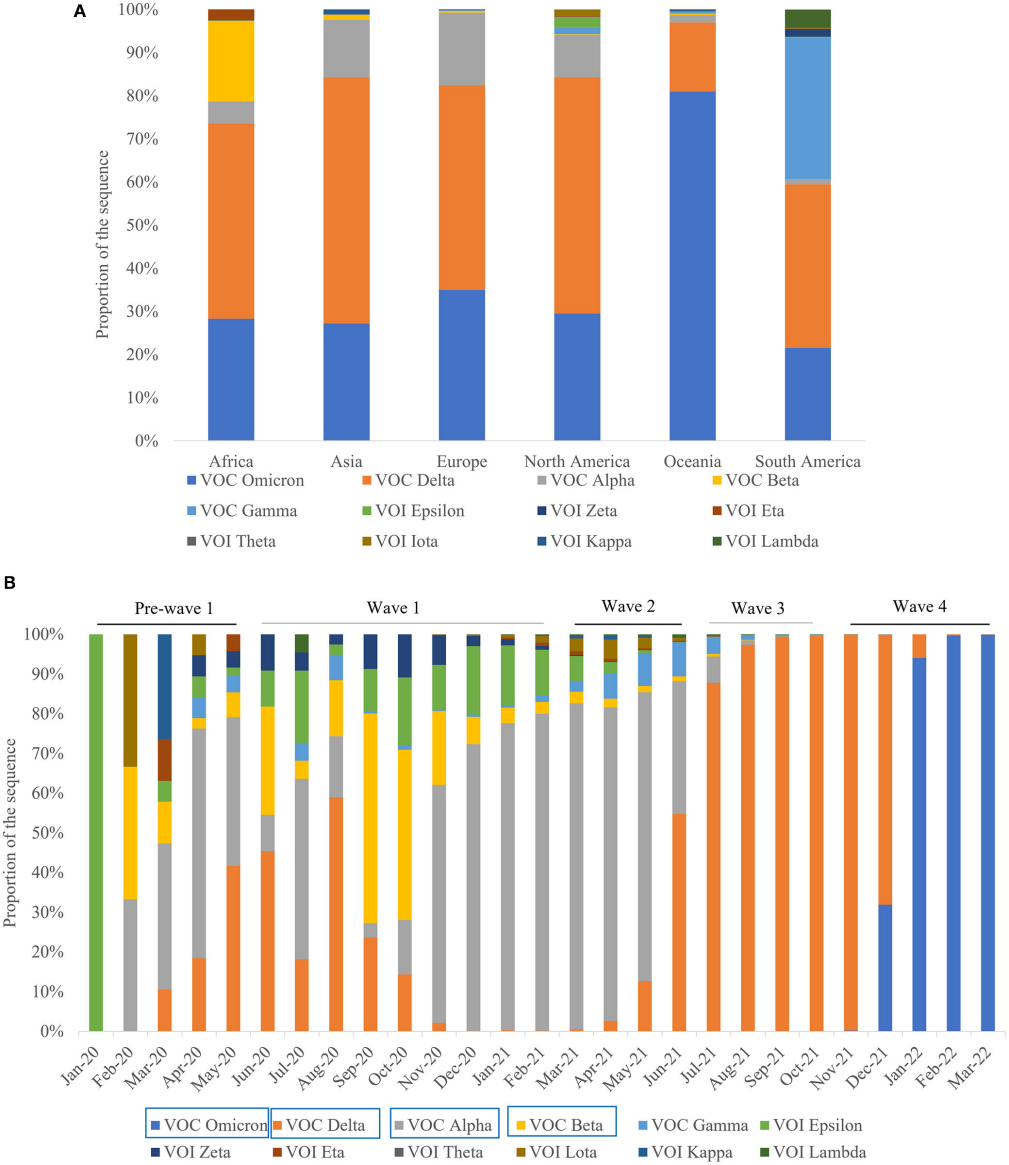
Diversity and distribution profile of SARS-CoV-2 variants. **(A)** Diversity profile of SARS-CoV-2 variants in the six continents. **(B)** Spatiotemporal distribution characteristics of the 12 SARS-CoV-2 variants among the four waves of the pandemics. Note: The Variants marked by blue rectangular boxes represent the predominate variants.

By March 31, 2022, 8,358,642 SARS-CoV-2 genomes had been submitted to the GISAID database from six continents worldwide (https://www.gisaid.org/). Furthermore, 12 SARS-CoV-2 variants were observed in six continents which included VOC Omicron, VOC Delta, VOC Alpha, VOC Beta, VOC Gamma, VOI Epsilon, VOI Zeta, VOI Eta, VOI Theta, VOI Iota, VOI Kappa, and VOI Lambda in Africa, Asia, Europe, North America, and Oceania; 11 variants, except VOI Theta, were observed in South America (Figure 3A; https://www.gisaid.org/). Among these, VOC Delta and VOC Omicron variants were the most frequently identified lineages in the six continents, while VOC Gamma was the most frequently identified lineage in South America (Figure 3A; https://www.gisaid.org/). The distributions of SARS-CoV-2 variants showed an obvious spatiotemporal change in the four pandemic waves. In the first wave, the main VOCs were Alpha, Delta, Epsilon, and Beta. VOC Alpha and VOI Epsilon were the dominant lineages in the second wave, while VOC Alpha and VOC Delta variants were mainly recorded in the third wave. In the fourth wave, Delta and Omicron were the predominant variants (Figure 3B). As the COVID-19 pandemic continues, the types of SARS-CoV-2 variants shift from multiple variants driving spread to a single lineage fueling spread.

### COVID-19 vaccinations worldwide

As of March 31, 2022, 64.6% of the global population had received at least one dose of the COVID-19 vaccine, with 11.33 billion doses administered globally, and 15.55 million administered daily. Only 14.7% of people in low-income countries had received at least one dose. By April 13, 2022, the cumulative COVID-19 vaccinations per 100 people were 145.07 doses worldwide, 33.89 doses in Africa, 167.47 doses in Asia, 167.15 doses in Oceania, 190.08 doses in South America, 161.94 doses in North America, and 168.24 doses in Europe. From December 13, 2020, to April 13, 2022, the total number of people who received all doses prescribed by the initial vaccination protocol reached 4.60 billion globally, 3.18 billion in Asia, 489.66 million in Europe, 374.53 million in North America, 319.04 million in South America, 211.21 million in Africa, and 27.30 million in Oceania. The percentage of people who received at least one vaccine dose, divided by the total population of each continent was 64.80%, while that in Africa, Asia Europe, North America, Oceania, and South America were 20.50, 74.70, 68.30, 71.60, 66.30, and 83.70%, respectively (Supplementary Figure S2).

## Discussion

In this study, we conducted a preliminary summary to examine the positive COVID-19 cases, deaths, VOC diversity, and vaccinations at the global level. Our analysis shows that there was a substantial difference in the evolving trend of COVID-19 in the six continents. Our analysis showed that the highest total cases per million and total deaths per million were observed in Europe and South America, respectively, despite these developed countries having higher rates of vaccination compared to other continents, such as Africa. However, both the total cases and deaths per million in Africa were the lowest. These data revealed that the vaccine currently used in these continents cannot completely prevent the spread of COVID-19. Similarly, a recent report suggested that vaccination may not completely prevent SARS-CoV-2 infection and stop its transmission, but it can prevent the occurrence of post-infection disease or reduce the severity of the disease (15). Meanwhile, high income levels likely increase the mobility and number of contacts, which increases the rate of new cases and deaths (16). In addition, traveling and population dispersal can aggravate the spread of disease in each region (17, 18). Although air travel has clearly been a major driver of the pandemic, intercontinental travel restrictions were less effective between Europe and the USA (19). Certainly, this is one of the most reasonable explanations for the highest number of cases and deaths reported in these continents. In addition, these continents had robust COVID-19 testing capacities and effective public surveillance systems, which are crucial to preventing a high infection rate. Approximately 3.4 billion tests were performed globally from December 2019 to August 2021, most of which were restricted to high-income countries, which conducted more SARS-CoV-2 testing (i.e., USA: 192%, Australia: 146%, Switzerland: 124%, and Canada: 113%) compared to that undertaken in low-income countries (LICs; i.e., Bangladesh: 6%, Uganda: 4% and Nigeria: 1%) (20). Furthermore, several other factors, such as the fragile healthcare system, relatively low population density, low obesity or diabetes burden, younger population, climate, genetics, and lessons from the public health response to other deadly infectious diseases (e.g., Ebola virus) are correlated with the lower case and death rates observed in Africa (21–23). Given the selective advantage offered by malaria through a wide distribution of blood group O in Africa, the enormous spread of the angiotensin-converting enzyme II (ACE2) deletion among many African ethnic groups might have reduced COVID-19 susceptibility in Africans (24). Likewise, countries with very low GDPs were less affected by the COVID-19 pandemic (16). This may be because low incomes limit the mobility of the population, thereby reducing the number of contacts with infected people. Additionally, there is a clear underreporting of the total burden of COVID-19 in Africa due to the undetected and insufficient death registration capacities (8). Finally, because of limited test capacity, the focus is on the observation of symptoms in individuals; however, there is a limited capacity for control when targeting symptomatic individuals, especially given that 65%−85% of COVID-19 cases in Africa do not present any symptoms (25). International efforts to assist undeveloped countries (such as Africa) are needed to support the global COVID-19 response (26), which should be followed by increasing the test per case ratio to gain an accurate understanding of the COVID-19 situation in this continent.

Our analysis showed that there were at least four waves of the COVID-19 pandemic globally, as well as in each continent, during the period examined. Although the number of cases in the fourth wave exponentially increased compared to those in the first wave, approximately 43.1% of deaths were in the first wave; this was due to multiple factors, including the health emergency in the initial stage, gradual reduction in the severity of novel variants, and the protection conferred by prior infection and/or vaccination. At the initial stage of the pandemic, when confronting COVID-19, there was less experience, a lack of relative knowledge, and no preparedness to fight against it. These factors led to a delayed response which then prolonged the pandemic period. With the prolongation of the pandemic, global governments pursued proactive measures, including the use of facemasks, hand sanitizers, lockdowns, increasing testing, contact tracing, and the roll-out of vaccines, which may explain why the second and third waves were shorter than the first wave. Although many developed countries have high rates of vaccination, the number of cases surged suddenly in the fourth wave, implicating that the driving factors of COVID-19 have the potential to induce significant changes; furthermore, the current vaccines used in these continents cannot completely prevent the spread of disease.

Meanwhile, since the pandemic, SARS-CoV-2 has dramatically evolved into numerous variants with an increase in transmissibility characteristics (27, 28). There have been at least 12 variants observed among the six continents, which showed a clear spatiotemporal profile. Likewise, multiple genetic lineages have been shown to co-circulate, although four were predominant at different periods in Barcelona city (Catalonia, Spain). Moreover, while B.1.5 (50.68%) and B.1.1 (32.88%) were the major lineages during the first pandemic wave, B.1.177 (66.85%) and B.1.1.7 (83.80%) were predominant during the second, third, and fourth waves (29). In the first wave, the main VOCs were Alpha, Delta, Epsilon, and Beta, while VOC Alpha and VOI Epsilon were the dominant lineages in the second wave. Alpha is not only more transmissible than pre-existing SARS-CoV-2 variants, but may also cause more severe illness (30). Furthermore, the Beta and Delta variants have a higher risk of spreading than the Alpha and Gamma variants (31). Generally, viruses mutate to adapt and sustain themselves in the environment, and the mutation causes an increase in transmissibility and the neutralizing capacity of the virus (32). The Delta variant was diagnosed in 51%−67% more cases than the Alpha variant and was also associated with higher hospital admission and emergency care attendance risk for patients with COVID-19 (33). In Qatar, a study showed that infection with the SARS-CoV-2 Delta variant was associated with more severe disease than infection with the Beta variant (34). From April 27, 2021, to September 12, 2021, 601,349 cases and 15,018 deaths were reported in Vietnam caused by the Delta variant, which was confirmed as the most complicated and dangerous variant, with the most deaths recorded (35). This may explain the highest number of deaths observed in the first two waves. Although VOC Alpha and VOC Delta variants were mainly recorded in the third wave, the lowest number of deaths in the third wave was considered to be mostly due to the protection conferred by prior infection and/or vaccination. For example, the SARS-CoV-2 seroprevalence in Slovenia increased four-fold from late April to October/November in 2020, mainly due to the emergence of a devastating second wave (36). In the fourth wave, Delta and Omicron were predominant, followed later by only Omicron, which subsequently caused the infection to increase sharply. The VOCs, Alpha, Beta, Gamma, and Delta, have a closer genetic relationship among themselves and a more distant genetic relationship with the Omicron variant (37). Omicron has greater transmissibility and infectivity, as well as an improved ability to evade immunity established by natural infections or vaccination (38). When a new variant with higher transmissibility and lower vaccine efficiency emerges, it becomes the dominant circulating variant. At present, Omicron has a significant growth advantage over the Delta variant, and also spreads more rapidly; indeed, in countries with known community transmission, it has a doubling period of 1.5–3 days (39). According to month, the highest case numbers (*n* = 89,374,078) were reported in January 2022. Similar pandemic waves caused by the Omicron were registered in January 2022 in the highly vaccinated UK, USA, and EU, potentially due to the lifting of quarantine restrictions for vaccinated people in these countries (40). Record numbers of new cases registered in late 2021 and early 2022 once again proved that existing vaccines cannot prevent new infections, and that vaccinated people can spread the infection as intensively as non-vaccinated ones (41). However, the prevalence of symptoms that characterize an Omicron infection differs from those of the Delta SARS-CoV-2 variant, which appears to be associated with reduced involvement of the lower respiratory tract and reduced probability of hospital admission (42). Although Omicron spreads significantly faster than the Delta variant (and other variants), it causes less severe disease (43–45). This result is in line with our analysis that surge and numerical cases were caused by Omicron but associated with fewer deaths (46, 47). Similarly, the trend of increasing cases and admissions across South Africa's first three waves shifted in the Omicron wave, with a higher and more rapid peak, but with fewer hospital admissions, less clinically severe illness, and a lower case-fatality ratio than the three preceding waves (48). Although the COVID-19 case peak growth was 18.6% higher than that during the Delta outbreak period in South Africa, the growth in death trends in the Omicron outbreak period was low, possibly due to the low mortality rate and case fatality proportion (49). Therefore, mild infectious groups account for most of the infected individuals, and prompt immunization results in weaker pandemic waves across all levels of infection, as well as a lower number of disease-caused deaths (50). Our analysis confirmed that the driving dynamic in the COVID-19 pandemic has changed and that the shift from multiple SARS-CoV-2 variants to a single lineage is responsible for driving the global pattern of multiple waves. Thus, there is an urgent need for continuous surveillance of variants of circulating lineages to reduce the further spread and to better understand the pandemic dynamics (51).

Currently, SARS-CoV-2 pandemics remain a global issue; two new Omicron variants were identified recently in South Africa, driving a surge in COVID-19 cases (52, 53). Furthermore, Omicron subvariants BA.4 and BA.5 have together become dominant variants in the USA (54). Importantly, both BA.4 and BA.5 are spreading faster than other Omicron lineages and can circumvent some immune protection conferred by previous infection and vaccination (55, 56). Local, national, and international health agencies have advocated multi-pronged public health strategies to limit infections and prevent deaths (57). Although the number of fully vaccinated people per 100 in six continents is not balanced across the six continents studied (58), mitigation measures such as masking and social distancing will not fully prevent transmission of the Omicron variant but will reduce the pressure on health systems worldwide. Moreover, vaccinations can significantly reduce the likelihood of deaths, and the increase in the number of tests per case ratio diminishes the number of infections (41). Furthermore, based on public strategies, restoring quarantine restriction populations (both vaccinated and non-vaccinated) and increasing the number of tests per case ratio is the optimal strategy for controlling the COVID-19 pandemic (40).

The current study has several limitations that warrant discussion. First, a simple definition of “pandemic wave” based on the number of increasing and decreasing cases may not fully reflect the dynamic profile of the pandemic. For instance, if the changes in the pandemic dynamics can be called “waves,” then there were five global waves predicted in 2020 alone (59). Second, our study focused on conducting statistical analyses without empirical models; however, a dynamic model analysis for quantitative interpretation of the relationships between variables could better grab the dynamic and epidemiological profile of the COVID-19 pandemic (60, 61) and assist in implementing measures to reverse pandemic trends.

## Data availability statement

The original contributions presented in the study are included in the article/Supplementary material, further inquiries can be directed to the corresponding authors.

## Author contributions

LG and ZhiL performed the data collection and processed and drafted the manuscript. ZhiL, XD, and ZheL participated in the design of the study. QS and KX prepared and critically reviewed the manuscript. CZ and LW participated in the study design and managed the project. All the authors have read and approved the final manuscript.

## Funding

This work was supported by the National Key R&D Program of China (Grant Nos. 2020YFE0205700, 2019YFC1200700, 2019YFC12006016, and 2021YFC2401000), the Youth Science Foundation of the State Key Laboratory of Infectious Disease Prevention and Control, China CDC (Grant No. 2021SKLID503), the National Natural Science Foundation of China (No. 82073624), and China-Sierra Leone Biosafety Laboratory Technical Cooperation Project (III Phase).

## Conflict of interest

The authors declare that the research was conducted in the absence of any commercial or financial relationships that could be construed as a potential conflict of interest.

## Publisher's note

All claims expressed in this article are solely those of the authors and do not necessarily represent those of their affiliated organizations, or those of the publisher, the editors and the reviewers. Any product that may be evaluated in this article, or claim that may be made by its manufacturer, is not guaranteed or endorsed by the publisher.

## Supplementary material

Supplementary Figure S1 The changes trends of Rt value in six continents over time.

Supplementary Figure S2 Percentage profile of people who received at least one vaccine dose in the six continents.
