# Supplementary figures and images for: Evolving trend change during the COVID-19 pandemic
Source: Front Public Health. 2022 Sep 20;10:957265. doi: 10.3389/fpubh.2022.957265 (PMC9531778; doi:10.3389/fpubh.2022.957265)

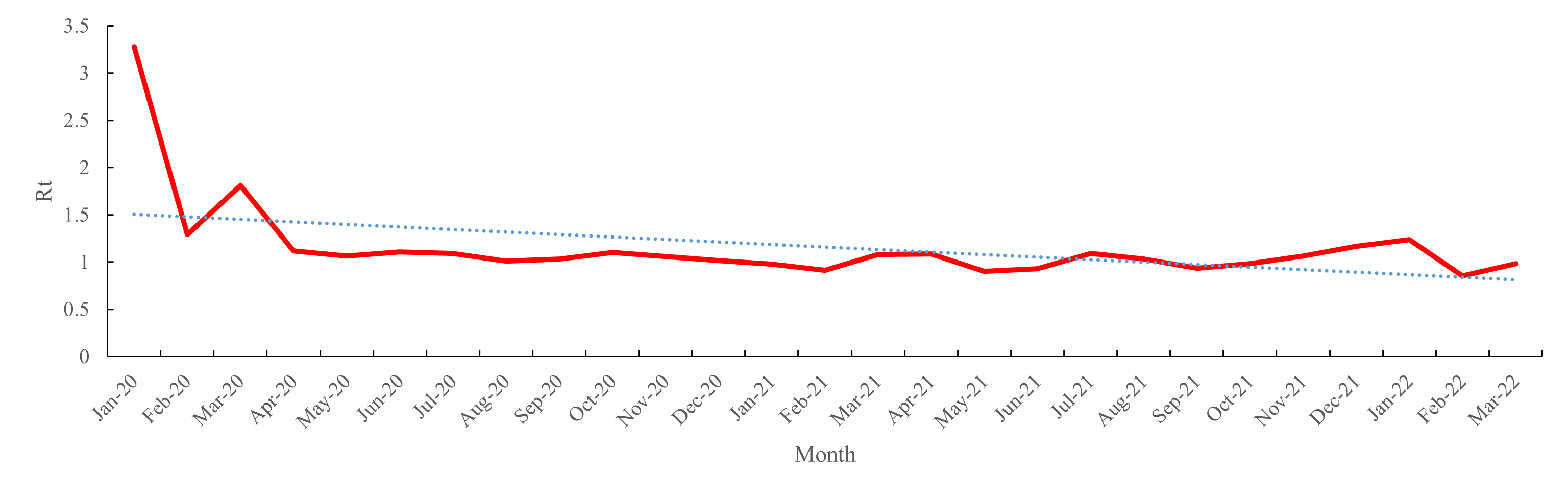

Supplement: Supplementary Figure S1 — The changes trends of Rt value in six continents over time. [file Image_1.tif]

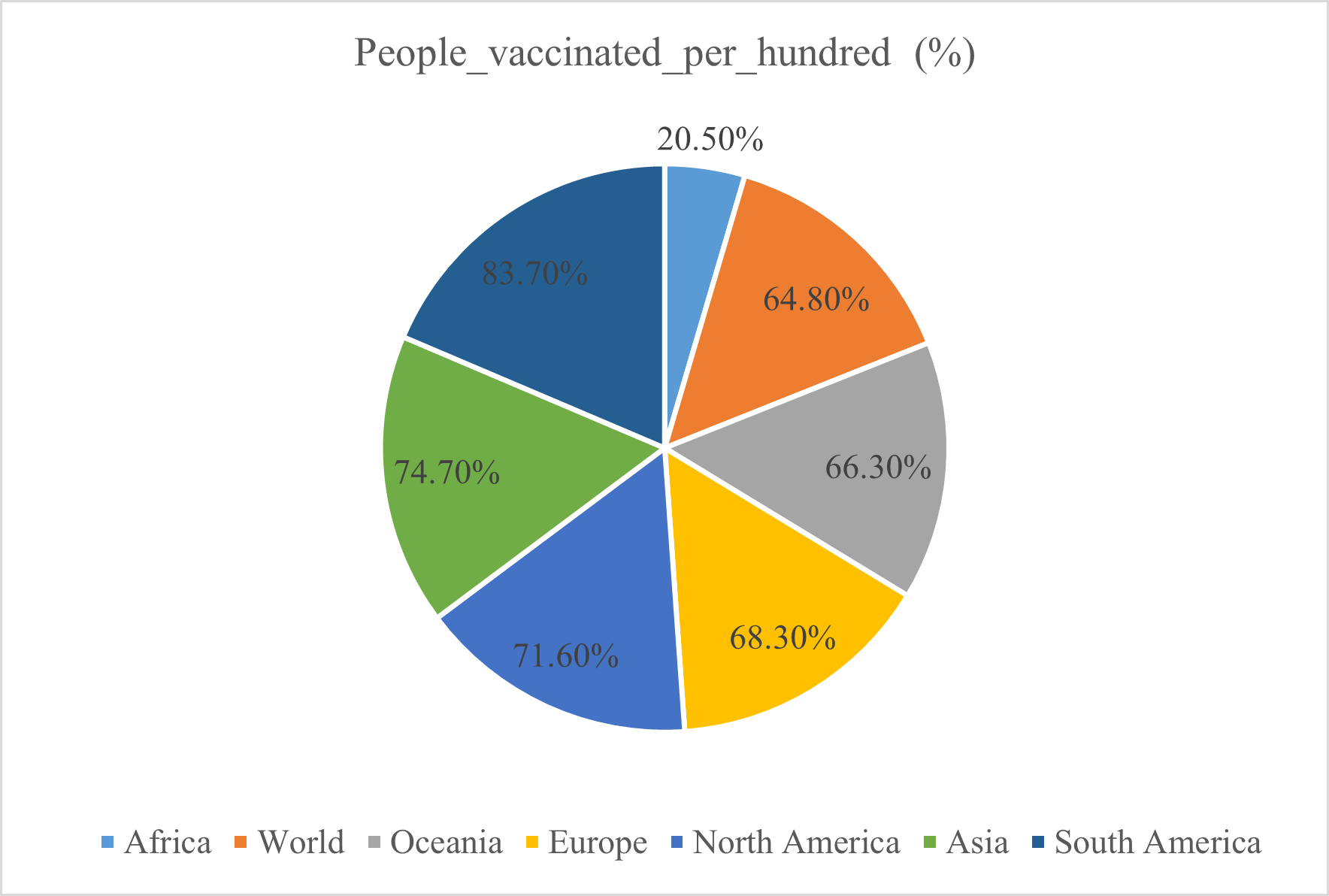

Supplement: Supplementary Figure S2 — Percentage profile of people who received at least one vaccine dose in the six continents. [file Image_2.tif]
