# Supplementary material for: Evolving trend change during the COVID-19 pandemic
Source: Front Public Health. 2022 Sep 20;10:957265. doi: 10.3389/fpubh.2022.957265 (PMC9531778; doi:10.3389/fpubh.2022.957265)
Supplement: Supplementary file 3 [file Table_1.DOCX]

| **Country** | 2020/3/14 | 2022/3/31 | Absolute Change | Relative Change |
| --- | --- | --- | --- | --- |
| **Africa** | 2.78% | 2.18% | -0.6 | -22% |
| **Asia** | 3.74% | 1.00% | -2.74 | -73% |
| **Europe** | 3.85% | 0.98% | -2.86 | -74% |
| **Oceania** | 1.16% | 0.16% | -1 | -86% |
| **North America** | 1.79% | 1.50% | -0.3 | -17% |
| **South America** | 2.06% | 2.30% | 0.24 | 11% |
| **World** | 3.71% | 1.25% | -2.46 | -66% |

Table S1 The case fatality rate (CFR) of six continents
